# An analysis of the prevalence of peripheral giant cell granuloma and pyogenic granuloma in relation to a dental implant

**DOI:** 10.1186/s12903-021-01566-4

**Published:** 2021-04-23

**Authors:** Nieves Román-Quesada, Beatriz González-Navarro, Keila Izquierdo-Gómez, Enric Jané-Salas, Antonio Marí-Roig, Albert Estrugo-Devesa, José López-López

**Affiliations:** 1 Faculty of Medicine and Health Sciences (Dentistry), University of Barcelona, Barcelona, Spain; 2 Department of Odontoestomatology, Faculty of Medicine and Health Sciences (Dentistry), Bellvitge Campus, University of Barcelona, Barcelona, Spain; 3 Oral Health and Masticatory System Group, Institut D’Investigació Biomédica de Bellvitge (IDIBELL, Bellvitge Institute of Biomedical Research), L’Hospitalet de Llobregrat, Barcelona, Spain; 4 Department of Maxillofacial Surgery, Bellvitge University Hospital, L’Hospitalet de Llobregrat, Barcelona, Spain; 5 Odontology Hospital University of Barcelona (HOUB), Barcelona, Spain

**Keywords:** Dental implant, Oral implant, Pyogenic granuloma, Peripheral giant cell granuloma, Reactive oral lesions

## Abstract

**Background:**

The aim of the present investigation was to evaluate the literature recurrence of peripheral giant cell granuloma and pyogenic granuloma associated with dental implants. It’s important to know the characteristics present in these lesions and possible effects on the prognosis of dental implants.

**Methods:**

An electronic search without time restrictions was done in the databases: PubMed/Medline. With the keywords "Granuloma" OR "Granuloma, Giant Cell" OR "peripheral giant cell" OR "Granuloma, Pyogenic” AND "Dental implants" OR "Oral implants”.

**Results:**

After applying the inclusion and exclusion criteria, a total of 20 articles were included, which reported 32 lesions (10 pyogenic granulomas, 21 peripheral giant cell granulomas and one peripheral giant cell granuloma combined with peripheral ossifying fibroma, all associated with implants). According to our review, these lesions are more frequent in males and in the posterior region of the mandible. Both excision and curettage of the lesion, compared to only excision, presented similar recurrences (40%). Explantation of the implant was performed in 41% of cases without additional recurrences. The results are not statistically significant when comparing one lesion to the other in terms of explantation (*p* = 0.97), recurrence (*p* = 0.57) or bone loss (*p* = 0.67).

**Conclusions:**

The main therapeutic approach is tissue excision. The lesions show a high recurrence rate (34.4%), which often requires explantation of the associated implant. This recurrence rate is not affected by curettage after excision.

## Background

The replacement of missing teeth with dental implants has a high success rate, but it is still a technique which involves risks and requires good evaluation and planning to minimize failures [1, 2]. However, the increasing number of dental implants used leads the dentist to encounter biological and technical complications [3, 4]. The most prominent biological complications are peri-implant mucositis and peri-implantitis [3, 5, 6]. In a 2017 systematic review, Lee et al. [7] demonstrated that the rates of mucositis and peri-implantitis were 29.48% and 9.25%, respectively.

The appearance of so-called peri-implant reactive lesions is another complication that must be taken into consideration. Despite its lower incidence, its presence could imply the need for explantation [2, 3, 8]. Reactive lesions are characterized by excessive proliferation of connective tissue in response to chronic irritation [9]. Among these lesions, those most frequently observed in the oral cavity are pyogenic granuloma (PG), traumatic fibroma, fibroepithelial hyperplasia, peripheral ossifying fibroma and peripheral giant cell granuloma (periheral giant cell lesion, -PGCG-) [4, 9]. PG and PGCG are the reactive lesions most frequently associated with teeth and implants [2, 9]. Some factors such as chronic inflammation, the accumulation of foreign bodies or corrosion of the implant surface could cause a chronic irritative process and act as contributing factors not only for mucositis and peri-implantitis but also for PG and PGCG [3, 4, 10].

**Fig. 1:**
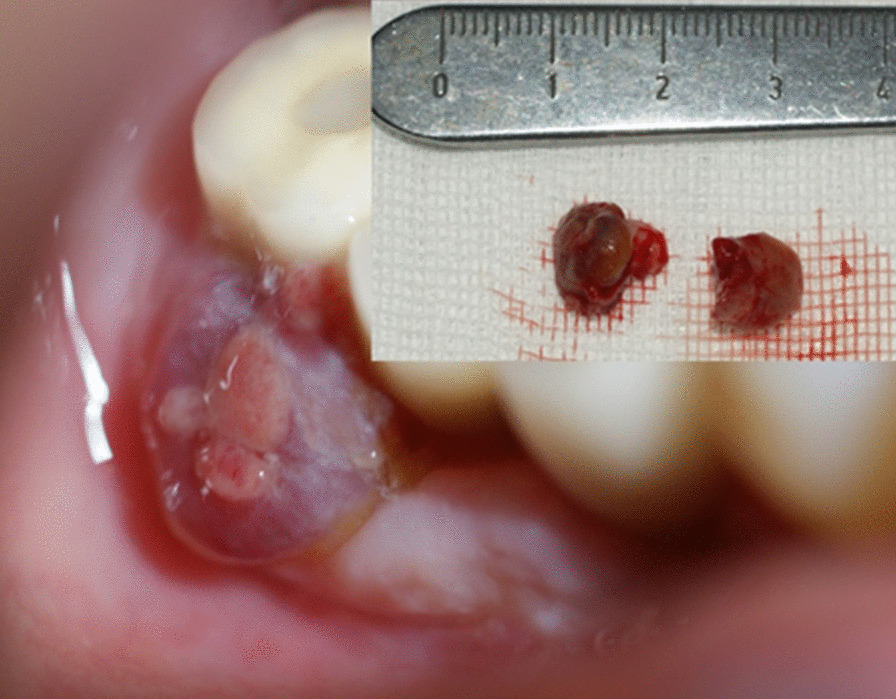
Pyogenic granuloma associated with a dental implant

Several factors have been studied that could interfere with osseointegration and therefore the survival of the placed implant. Factors such as smoking, diabetes and periodontal disease have been studied [9, 11]. However, with regard to reactive lesions, such as PG and PGCG, the prevention technique is the maintenance of healthy peri-implant tissue [9] (Fig. 1).

PG can occur in response to irritants, trauma, hormonal changes or certain medications. Although classically called PG, the most correct name would be capillary lobular hemangioma, since the lesion is not strictly a granuloma or an infection [9, 10]. Clinically, oral PG is characterized as an exophytic mass with a smooth or lobulated appearance that can be sessile or pedunculated. The epithelium is frequently ulcerated and varies in color from pink to red and/or purple, depending on the evolution time and the vascularization of the lesion. The lesion tends to grow rapidly in a short period of time, with a tendency to bleed spontaneously or after a minor trauma [10, 12]. Its incidence varies between 3.81 to 7% of the histopathological results of biopsies performed in the oral cavity [3, 12]. According to the literature, it is more frequent in young women (3:2), more common in the maxilla than in the mandible, and in the anterior areas when associated with teeth [9]. Histologically, PG is composed of proliferating blood vessels and granulation tissue, often organized in lobular aggregates, hence the name capillary lobular hemangioma [3]. It is characterized by prominent capillary growth in hyperplastic granulation tissue, suggesting high angiogenic activity. Blood vessels often show a grouped or separated pattern by less vascular fibrotic septa, leading some authors to consider PG as a polypoid form of capillary hemangioma [12].

PGCG is believed that its pathogenesis includes an excessive activation of osteoclasts, which is associated with a proliferation of macrophages and can cause significant bone resorption [9]. Clinically, PGCG may present as a firm or soft, sessile or pedunculated nodule, color ranging from bluish to purple, with a frequently ulcerated surface, confined to the alveolar and/or gingival mucosa. Swelling is the most frequent sign and its clinical course is usually asymptomatic [4, 13]. It tends to show a progressive increase in size and can cause pressure on the adjacent teeth, which could lead to malocclusion or interference with mastication. Erosion of the underlying bone or periodontium can also occur and in edentulous areas, it can often cause a radiolucent cup-shaped image in intraoral radiography [4]. PGCG does not have an age predilection. It seems to have a greater predilection in women and tends to appear more frequently in the molar area [4, 9, 11]. It consists of a non-encapsulated lobulated tumor of proliferating vascular connective tissue with a large number of osteoclast-type multinucleated giant cells. It is common to find signs of bleeding and hemosiderin deposits inside the lesion. Fibroblastic proliferation and/or significant formation of osteoid material and even bone is common. Blood biochemistry shows no alterations [3, 13]; however, if alteration of phosphorus-calcium metabolism appears, hyperparathyroidism should be suspected and a brown parathyroid tumor should be ruled out [14–16].

The treatment of both PG and PGCG includes complete surgical excision until bone level and complete curettage, as well as removal of the causative agent if identified [17]. Since it is rarely reported it’s important to know the characteristics present in these lesions and possible effects on the prognosis of dental implants in order to take the appropriate course of action in the clinical practice.

The aim of the present study was to search the association of the available data published in the literature on PG and PGCG associated with dental implants, analyzing their recurrences, associated etiological factors and different treatment options. For this we asked ourselves the following PICO question: What is the prevalence, recurrence, etiological factors and treatment of PG and PGCG around dental implants? P: Dental implants; I: development of a reactive lesion; C: PG vs. PGCG, type of treatment carried out (excision vs. excision and curettage); O: Prevalence of PG and PGCG around dental implants, recurrence of the lesions and bone loss around dental implants associated with the lesions.

## Methods

### Search strategy

The present study followed the Preferred Reporting Items for Systematic Reviews and Meta-Analysis (PRISMA) guidelines [18]. An electronic search was conducted in February 2020, with no time restrictions in the database: PubMed/Medline. The following terms were used for the search strategies: ["Granuloma" OR "peripheral giant cell granuloma" OR "Granuloma, Giant Cell" OR "peripheral giant cell" OR "Granuloma, Pyogenic" OR "peripheral giant cell reparative granuloma” OR "peripheral giant cell epulis”] AND ["Dental implants" OR "Oral implants”].

### Article selection and inclusion criteria

The inclusion criteria were publications on cases of PG and PGCG associated with dental implants, with sufficient clinical, radiological and histological information to confirm the diagnosis (at least 8 out of the 10 outcomes of the table should be included); case series and single case studies. Articles that were written in English or Spanish. Articles with other lesions or syndromic conditions with similar characteristics, review, book chapter and letter to the editors were excluded.

### Data extraction and analysis

For each of the included studies, the following data were extracted (whenever available): age, sex, location of the lesion, implants involved, presence of bone loss, time between implant placement and the appearance of the lesion, treatment performed, recurrence, implant failure and possible associated pathologies observed. Regarding bone loss, we made a subjective assessment; classifying severe as exposure of more than 3 implant threads or bone loss of ≥ 4 mm, moderate to less than 3 exposed threads (between 3–4 mm) and mild as a loss of less than 3 mm of bone based on radiographs and intraoral photographs or on the authors' criteria.

All cases are initially selected and analysed by NRQ & BGN, discrepancies are consulted with EJS, KIG & AMR and in case of doubt they are finally validated by AED and JLL.

All statistical analyses were conducted using Statistical Package for the Social Sciences (SPSS 12.0, Chicago, IL). Mean values of age and time of diagnosis of the lesions were estimated. Fisher’s exact test was used to analyze recurrence of lesions and bone loss. All reported values (P values) were compared to a significance level of 5%.

## Results

**Fig. 2:**
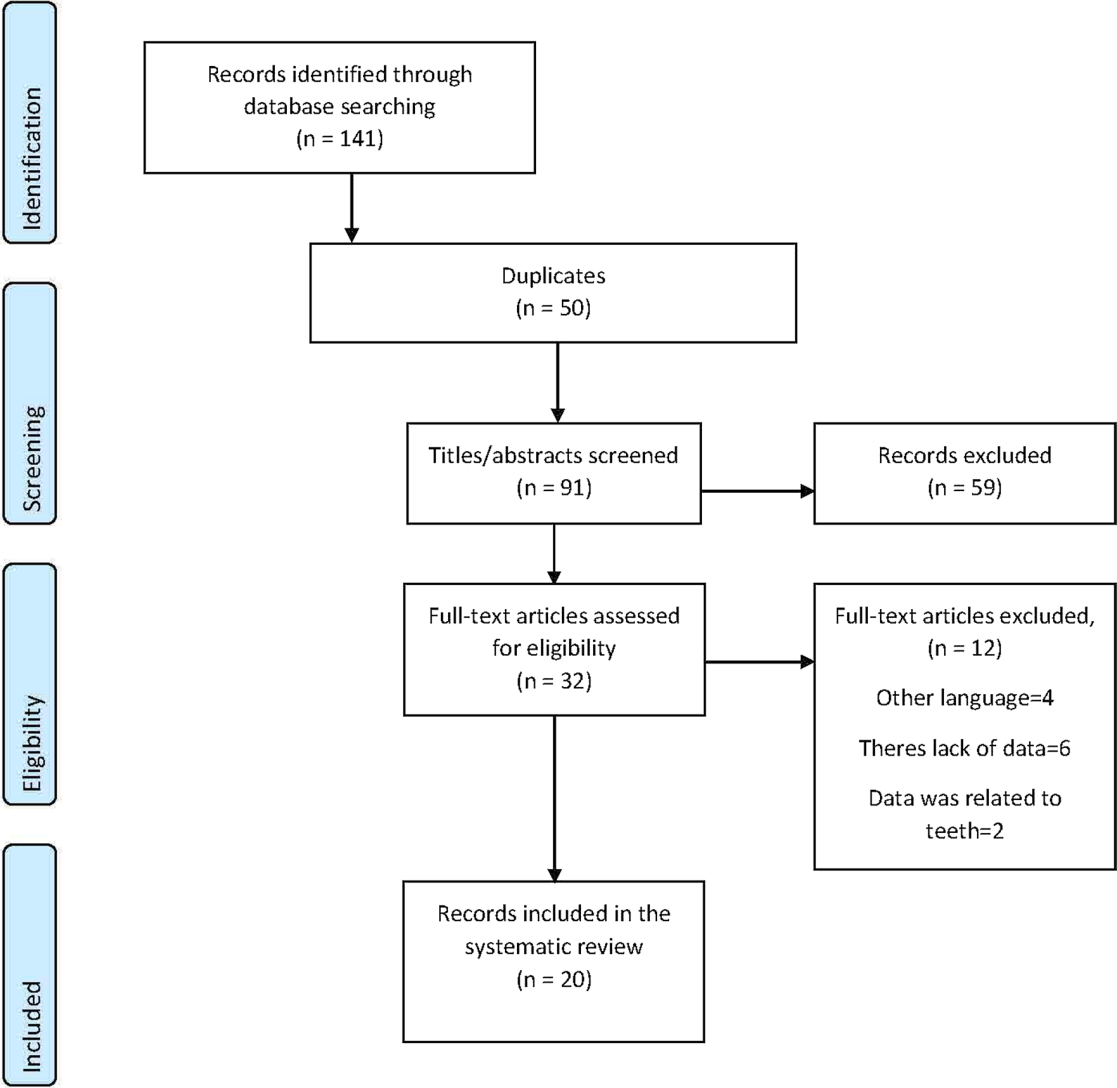
PRISMA flowchart

141 articles were obtained with the search strategy used. After excluding duplicate articles, 91 articles were selected and reading the title and abstract, 32 were left. Out of 32 articles, 4 were excluded because they were not written in English or Spanish, 6 due to lack of data and 2 because data was related to teeth instead of implants. Finally, 20 articles that met the inclusion and exclusion criteria previously specified were selected (Fig. 2).

The 20 papers analyzed [4, 9, 11, 17, 19–34] reported 30 patients with 32 lesions around 51 implants involved, and 2 patients reported two lesions. Histologically, 10 lesions were diagnosed as PG [9, 22, 24–26, 28, 30, 31], 21 as PGCG [4, 9, 11, 17, 19–21, 23, 29, 32–34] and one combined PGCG with an ossifying peripheral fibroma [24]. The epidemiological and clinical characteristics are summarized in Table 1.

**Table 1:** Summary of the most important characteristics of PGCG and PG associated with dental implants

References	NP	Sex	Age	Type of lesion	Loc	Appearance Time	No. of Imp	Bone Loss	1º Treatment	2º Treatment	Recurrence/follow up	Observation
Hirshberg et al. [23]	3	♂	31	PGCG	Md post	months (*)	1	Mild	Excision + curettage	Excision with Arg laser	1º. 1 2º. No	NR
		♂	69	PGCG	Mx ant	14 months	1	Mild	Curettage	Excision with laser + exp	1º. 1 2º. No	Mild congestive cardiac insufficiency
		♂	44	PGCG	Md post	6 years	1	Mild	Excision + curettage	Excision + curettage + exp	1º. 3 2º. No	NR
Cloutier et al. [21]	1	♂	21	PGCG	Md post	6 years	2	Severe (+ in 4.6)	Excision + exp (4.6) + curettage	–	1º-No	NR
Hernandez et al. [11]	3	♀	45	PGCG	Md post	3 years	2	Severe	Excision + curettage	Excision + exp + curettage	1º. 5 2º. No	NR
		♀	36	PGCG	Mx post	2 years	1	Severe	Excision + curettage + CLX	Excision + exp + curettage	1º. 4 2º. No	Calculus remains
		♀	62	PGCG	Md post	1 month	1	Moderate	Excision of the lesion	–	1º. No	Advanced chronic periodontitis
Olmedo et al. [24]	2	♀	75	PG	Md post	2 months	1	No	Excision + curettage	–	1º. No	NR
		♀	64	PGCG	Mx ant	12 years	3	Mild	Excision + curettage	–	1º. No	Broken screw in implant 21 Metallic particles
Dojcinovic et al. [25]	1	♂	32	PG	Mx post	6 months	1	No	Excision + curettage	–	1º. No	Sinus lift + autologous iliac crest graft
Peñarrocha-Diago et al. [19]	1	♀	54	PGCG	Md post	3 years	2	Moderate	Prosthesis removal + excision + curettage + implantoplasty	–	1º. No	Post extraction implants Prosthesis retention zone
Etöz et al. [26]	1	♀	55	PG	Md post	1 month	1	No	Excision + exp + curettage	–	1º. No	Xenograft® + PRF membrane
Kang et al. [22]	1	♂	68	PG + CH	Mx post	5 years	1	Severe	Excision + exp	–	1º. No	4 years after implant placement: infarct + warfarin
Jané-Salas et al. [9]	2	♂	52	PG	Mx post	3 years	2	Moderate	Excision + curettage + CLX	–	1º. No	Chronic periodontal disease + plaque deposition
					Md post	3 years	1	Moderate	Excision + curettage + CLX	–	1º. No	Chronic periodontal disease + plaque deposition
		**♂**	64	PGCG	Md post	8 years	1	Moderate	Excision + curettage + CLX	–	1º. No	Periodontal disease
Ogbureke et al. [27]	1	♂	44	PGCG + POF	Md post	3 months	2	No	Excision	–	1º. No	NR
Kaya et al. [28]	1	♂	34	PG	Mx ant	7 years	2	Moderate	Periodontal treatment + excision with laser	–	1º. No	Autogenous graft + alveolar osteogenic distraction
Brown et al. [20]	1	♂	46	PGCG	Md post	1 year	1	No	Excision	Excision + curettage	1º. 1 2º. No	NR
Pacifici et al. [29]	1	♂	60	PGCG	Mx ant	5 years	1	Severe	Excision + curettage	–	1º. No	Deficient oral hygiene
Truschnegg et al. [30]	1	♂	67	PG	Md ant	3 months post loading	2	Moderate (in 34)	Electrocauterization	Excision with laser + curettage + abutment change	1º. 2 2º. No	Deficient oral hygiene. Abutment in poor condition
Gefrerer et al. [31]	1	♀	55	PG	Md post	9 weeks	2	Severe	Excision + curettage	Exp Implant	1º. 4 2º. No	Autologous iliac crest graft
					Md post	1 year	2	Moderate	Excision + curettage	Exp Implant	1º. 2 2º. No	Autologous iliac crest graft
Galindo-Moreno et al. [32]	1	♂	74	PGCG	Mx ant	6 years	1	No	Bar removal + excision + CLX	–	1º. No	Periodontitis, ex-smoker (14 years) Bar retention zone
Scarano et al. [4]	3	♂	26	PGCG	Md ant	–	1	Severe	Excision + exp + curettage	–	1º. No	NR
		♀	52	PGCG	Mx post	–	1	Severe	Excision + exp + curettage	–	1º. No	NR
		♂	31	PGCG	Md ant	–	6	Severe	Excision + exp + curettage	–	1º. No	Deficient oral hygiene
Bidra et al. [17]	3	♂	49	PGCG	Mx ant	10 years	1	No	Excision + curettage	–	1º. No	Bone graft
		♀	64	PGCG	Mx ant	10 years	1	Severe	Excision + curettage	Exp + curettage	1º. 1 2º. No	Bone graft + deficient oral hygiene
		♂	65	PGCG	Md ant	2 weeks	4	No	Excision + curettage	Excision + curettage + fluocinolone + new prosthesis	1º. 1 2º. No	Post-extraction and immediate loading. Prosthesis with retentive zone
Baesso et al. [33]	1	♂	53	PGCG	Md post	–	1	Moderate	Excision + curettage	–	1º. No	Prosthesis with retentive zone
Mordini [34]	1	♀	39	PGCG	Mx ant	7 months	1	Mild	Excision + curettage	–	1º. No	Bone AloGraft + Trauma

NP: number of patients, Loc: location, No. of Imp: number of implants involved, PGCG: peripheral giant cell granuloma, NR: No reference; Md: mandible, post: posterior, PG: pyogenic granuloma, Arg: argon, Mx: maxilla, ant: anterior, exp: explantation, CLX: chlorhexidine, PG: pyogenic granuloma, PRF: platelet-rich fibrin, CH: capillary hemangioma, POF: peripheral ossifying fibroma, Exo: extraction, (*): Does not specify

Lesions were more prevalent (in this review) in men than in women (20:12), more in the mandible than in the maxilla, with a mean age of 51 years (range between 21 and 75 years). Information on the time between implant placement surgery and diagnosis of the lesion was available in 27 lesions with a mean of 3.5 years (range between 1 month and 12 years) [9, 11, 17, 19–32].

Regarding the first therapeutic approach, excision of the lesion was performed in all cases. In 5/32 cases [11, 20, 23, 27, 32], only the excision of the lesion was performed, presenting two cases of recurrence [20, 23]. In 19 out of 32 cases [9, 11, 17, 19, 23–25, 29, 31, 33] the treatment was accompanied by curettage of the underlying bone, with recurrences in 8 out of 19 cases [11, 17, 23, 31] and 6 of these 8 cases finally required explantation [11, 17, 23, 31] due to this recurrence and bone loss. One case [28] was treated using laser technique without recurrence and another [30] with electrocautery, which did recur. Finally, in the remaining 6 cases [4, 21, 22, 26], excision of the lesion was performed in association with the explantation, without later recurrence. Based on these data, we can say that of the 13 cases in which explantation was not performed, the rate of recurrence of the lesions surgically excised with curettage was not significant compared to only the removal of the lesion, with a *p* = 0.97. Due to the lack of data, we do not know if the lesions that were only eliminated were the smallest. According to these data, clinically, we would choose to excise the lesion without curettage, avoiding further bone loss and damaging the implant surface. However it should be taken into account that the patients sample who do not undergo excision plus curettage is significantly smaller than those who undergo curettage.

A total of 11 out of 32 (34.4%) lesions [11, 17, 20, 23, 30, 31] with recurrences were found, of which 4 [11, 23, 31] reported more than 3 recurrences, requiring an explantation to solve the issue. Of these 11 lesions, 9 were diagnosed as PGCG [11, 17, 20, 23] and 2 as PG [30, 31]. In this review, recurrence rate is not statistically significant for one lesion or another, with a *p* = 0.57.

In 75% of cases [4, 9, 11, 17, 19, 21–24, 28–31, 33, 34] bone loss was observed (Table 2). 10 cases (41.6%) reported severe [4, 11, 17, 21, 22, 29, 31], 9 cases (37.5%) reported moderate [9, 11, 19, 28, 30, 31, 33] and 5 cases (20.8%) reported mild bone loss [23, 24, 34]. Of the 24 cases with bone loss, implants involved were explanted in 45.8% [4, 11, 17, 21–23, 31]; of which 81.8% had severe bone loss [4, 11, 17, 21, 22, 29, 31] and 81.8% were PGCG [4, 11, 17, 21, 23] (Table 2). 8 cases (out of 32) did not present bone loss, comprising of 5 PGCG compared to 3 PG, and there was no statistical significance with a *p* = 0.65. If we correlate total severe bone loss [10, of which 8 are PGCG and 2 PG] with moderate or mild bone loss [14 of which 9 are PGCG and 5 PG], we also do not obtain significant results, with a *p* = 0.4.

**Table 2:** Distribution of cases with bone loss

	Bone loss				
	Yes			No	
	Severe	Moderate	Mild		
11	9*	1^#^	1^&^		Exp
21	1	8	4	8^$^	No exp
32	10	9	5	8	Total

There is no statistical significance

(*) 8 are PGCG and 1 is PG; (#) It is a PG; (&) It is a PGCG; Exp: Explantation; NO Exp: No explantation; ($) 5 are PGCG and 3 are PG

The attributed etiopathogenesis is varied (Table 3). In 71.8% (23 of 32) of the cases, observations that inform about a possible etiology were made. 26% (6 of 23) had poor oral hygiene, and 50% (3 of 6) required explantation. After the correction of these prostheses, there were no recurrences, and therefore explantation of the implants was not required. In 30% (7 of 23) of the cases, implant placement was performed with bone grafting, 57.1% (4 of 7) required explantation and in 2 of them (with associated PG) it is specified that iliac crest grafts were used [31], of the other 2, one presented PG and the other PGCG. Although in all 7 cases, there could be other associated factors, the authors preferred to attribute the occurrence to the graft. Finally, in 2 of the 23 cases the etiology was supposed to be periodontal disease [9, 11], both being PGCG and without explantation. One of the cases included in which the prosthesis was the cause of the lesion, was not included here because the author stated that the most relevant cause was the design of the prosthesis [17].

**Table 3:** Results of attributed etiopathogenesis

Attributed etiopathogenesis						
Bone graft	DOH	Prosthesis-PA-PA	Trauma	Adverse reaction to Warfarin	PD	
4*	3^&^	–	–	1^+^		Exp
3^#^	3^&^	6^$^	1^@^	-	2^++^	No exp
7	6	6	1	1	2	

DOR: Deficient Oral Hygiene; Prosthesis-PA-PA: Prosthesis Poorly adapted with plaque accumulation; PD: Periodontal Disease; Exp: Explantation; No Exp: No explantation

(*) 3 are PG and 1 is PGCG; (#) 2 are PG; (&) 2 are PG and 1 PGCG; ($) 5 are PGCG; (@) It is a PGCG; (+) It is a PG; (++) They are PGCG

## Discussion

PG and PGCG are inflammatory reactive lesions generally found around teeth, root stumps and dental implants. The main feature helping to differentiate PGCG from PG is the abundant presence of multinucleated giant cells [35]. Although PG is considered more frequent than PGCG as a lesion, in this review PGCG is more frequently reported than PG in association with dental implants, data also supported the study by Brown et al. [20]. Based on this review, implant-associated PG and PGCG are rare peri-implant soft tissue complications, initially reported by Hirshberg et al. [23] and as mentioned by Peñarrocha-Diago et al. [19], due to few documented cases, the etiology and incidence of these lesions are not well established.

With respect to location, both in our review and in the cases reported in the literature, PG and PGCG associated with implants have a predilection for the posterior regions of the jaws (20:12), mainly in the mandible (19:13) [9, 19]. This can be explained by the fact that the implants placed in the posterior regions hinder proper oral hygiene, which seems to be a factor that favors the appearance of these lesions [2, 9]. Although Hernández et al. [11] suggest that the occlusal forces experienced in the posterior area are also a risk factor for the development of reactive lesions around the implants. Or simply the fact that more implants are placed in these locations, as different authors report while analyzing the literature [2, 9, 31].

The literature refers that when these lesions (PG and PGCG) affect the peridental areas, they are more frequent in women, although some studies have shown an equal prevalence for both sexes [2, 9]. This is in contrast with our findings that suggest that when these lesions are associated with implants, they are more frequent in men (20:12). According to Woelber et al. [36], this finding could be due to the fact that men have a higher plaque index than women, probably due to a lower level of hygiene, however, this is an inconsistent conclusion.

Dental implants are most commonly used in elderly patients; therefore, reactive lesions are expected to appear in older patients compared to those seen around natural teeth [2, 20]. In this review, it was observed that the age range was from 21 to 75 years, with an average age of 51 years, similar to the studies by Atarbashi-Moghadam et al. [2] with a mean age of 51.28 ± 14.48 and Brown et al. [20] with an average age of 50.9 years.

A possible pathogenesis of PG and PGCG associated with dental implants is a chronic local irritation that acts on the gingival tissue. In this sense, the local irritating factors involved may include accumulation of plaque or calculus or the presence of foreign bodies, such as possible traces of dental cement or metal particles [2, 4, 19]. Burbano et al. [37] reported the presence of five types of dental cement particles in biopsies of peri-implantitis using scanning electron microscopy. Furthermore, Olmedo et al. [24] reported that the presence of metal particles in the tissues plays a role in the corrosion process of the metallic component of the implant. They proposed that macrophages capture these metal particles, stimulating the release of cytokines, which play a role in bone resorption by activating osteoclasts and suppressing osteoblast function, thereby reducing bone formation and promoting osteolysis. This deposit can occur during implant placement or due to the abutment connection [10, 20, 38]. Rodrigues et al. [39] supported this in a study that reported a PG and a PGCG associated with dental implants, and confirmed the presence of particles, possibly of titanium. However, the implant technique is common and there are few reference cases of PG and PGCG. This could possibly be influenced by the fact that some professionals surgically remove the peri-implant tissues and do not send them for a histopathological study [13], or simply do not publish the cases. An interesting study, without conclusive results, found some differences in microvascular density, proliferative activity, and CD68 and Bcl-2 expression, between conventional and implant-associated granulomatous lesions [40]. In this study, histological and immunohistochemical aspects of peri-implant granulomatous lesions were analyzed and 13 new cases were presented, 2 in men and 11 in women. Although this series is not included in this review due to the lack of individualized clinical data and not meeting our inclusion criteria, it is worth noting that the average age in this case series was 57.5 years, with a higher prevalence in women [2 of 13, 85%] and the posterior part of the mandible being the most affected area (10 of 13).

Peri-implantitis and marginal bone loss have been suggested to expose the unpolished portion of the implant neck, which can cause chronic irritation to the adjacent mucosa and lead to the development of reactive lesions [2, 13, 20]. Data from the articles selected in this review suggest that peri-implant bone loss is more commonly associated with PGCG than PG (17:7). Several authors [11, 21, 41, 42] support this hypothesis. In addition, Hirshberg et al. [23] in their review of 25 peri-implant biopsy samples indicate that the PGCC around dental implants can causes loss of crestal bone that can eventually lead to implant failure. Surgical excision down to the bone level and aggressive curettage can cause additional bone loss around the implant [17, 19]. Furthermore, Hernández et al. [11] have also speculated whether bone loss is the cause or consequence of the granuloma and believe that the higher prevalence of these lesions in the posterior regions may be due to the greater bone loss that occurs due to the greater implant overload caused by the occlusal forces. Data from the selected articles suggest that peri-implant bone loss is more commonly associated with PGCG; in the present study, 10 of the 24 lesions with the presence of bone loss were severe and 8 of these lesions were PGCG [9].

Bischof et al. [42] described a PGCG in a 56-year-old woman with three posterior mandibular implants. It seems that the angulation of the implants was inappropriate, which led to the healing abutments of two of them being poorly adjusted and juxtaposed. In addition, the patient reported the difficulty in cleaning the mouth, increasing the level of plaque in the area. This corroborates the study by Özden et al. [41], who described a case of PGCG associated with a dental implant that occurred due to a poorly fitted prosthesis, which led to the accumulation of dental plaque and irritation of the gums. Regarding poorly designed prostheses, Bischof et al. [42], Özden et al. [41], and Peñarrocha-Diago et al. [19] replaced the prostheses or temporarily removed it, which allowed better plaque control in these cases and the absence of recurrences. In our review, authors such as Bidra et al., Peñarrocha-Diago et al. and Baesso et al. [17, 19, 33] included removal of the prosthesis and replacement with a healing abutment, thus performing careful curettage and removal of the irritant factor; subsequently making a new prosthesis. We did not find justification in the literature to relate the appearance of the lesion to Warfarin medication, as suggested by Hirshberg et al. [23].

Günhan et al. [43] suggested that the appearance of these lesions could be due to the influence of sex hormones, since multinucleated giant cells are a target for estrogens. Immunohistochemical investigation of estrogen receptors in PGCGs turned out positive. However, it can be suggested that in the context of implant-associated PGCG, the hormonal influence would be less important, since most of the patients are postmenopausal and, therefore, serum estrogen levels are low [20]. Recurrences of PG and PGCG have been described, especially associated with teeth due to a hormonal imbalance during pregnancy, especially in cases of PG [9, 33]. Scarano et al. [35] article had as hypothesis that the multinucleated cells present in central giant cell granuloma of the jawbones may simply represent a reactive component of the lesion and is a response to stimuli from the stroma. These cells have been shown to present a positivity to estrogen and to show a phenotype different from that of other giant cells found in sites of chronic inflammation and may be true osteoclasts.

The length of time between implant placement and initial presentation of PGCG and PG in our review ranged from 1 month to 12 years. Although there is controversy, since tooth extraction has been described as an etiological factor for the development of PGCG, as demonstrated by Hirshberg et al. [23], who reported that the PGCG developed after tooth extraction in 8–11% of the cases examined, appearing up to one year after the procedure. Scarano et al. [35] also indicate that the occurrence of OGCG may be related to teeth extraction carried out before the implant insertion, Given this, surgical removal must be done otherwise the affected implant will fail. Therefore, it would be interesting to investigate whether the cases shown in Table 1 underwent a dental extraction before implant placement and, if so, the time between extraction and implant placement. In this sense, only Bidra et al. [17] and Peñarrocha-Diago et al. [19] specified that the implants were placed post-extraction [20].

The recurrence rate of PGCG associated with teeth is varied, but it has been shown to range from 15 to 20% in most studies compared to PG that ranges from 0 to 16% [2]. In our review, the recurrence rate for PGCG and PG associated with implants was 34.4%. Therefore, it appears that the recurrence rate of these implant-associated lesions is higher than those unrelated to them [9, 19]. The reason for this difference in recurrence rates is unclear, but it could be due to difficulty faced in the removal of peri-implant tissues. However, curettage after excision does not seem to affect the recurrence rate, since the results are not significant [2, 13].

Several treatments for implant-associated PG and PGCG have been described, with conservative surgical extirpation with total removal of the lesion base and bone curettage being the treatment of choice [2, 9, 20]. Any irritating factors that may be causing or promoting the appearance of PGCG and PG must also be eliminated. Hernández et al. [11] also recommend polishing the implant surface [19, 20]. Photodynamic therapy (PDT) has been used as a complementary treatment for peri-implant diseases. Its main objective is controlling disease progression through decontamination of infected surfaces. PDT is a simple and non-invasive technique that has proven to have antibacterial effects. Although further studies are needed to prove its efficiency in cases of PGCG around dental implants. [33]

Due to the aggressive nature of the lesion and the high recurrence rate, implants can fail unless the lesion is detected early and proper surgical removal is performed along with close follow-up. [23] The recommendation to remove an involved implant should be based on the clinical judgment of the practitioner balanced with the risk of recurrence of the lesion due to potential inadequate removal if access is jeopardized by the continued physical presence of a dental implant. [21]

The use of the Er-YAG, carbon dioxide or diode laser have been suggested as other treatment modalities and may offer advantages compared to conventional surgical techniques, especially by reducing the risk of postoperative bleeding, pain, and edema and also by eliminating the need to suture at the end of the procedure [2, 9].

In the study of Kaya et al. [28], to avoid a peri-implant reactive lesion, they decontaminated the exposed implant surface using the Er:YAG laser. There was no scarring or recurrence at the 2-year follow-up. Hence, an Er:YAG laser appears to be a good therapeutic option for intraoral pyogenic granulomas.

As limitations we have found that the works are clinical cases and the longest series of cases found is 3 cases. In many cases the incidence and etiology are not well established. Even in some reports the diagnosis is only clinical, which may represent an added bias. In some cases not all the data needed to perform the analysis is specified.

## Conclusions

Implant-associated granulomas are more frequent in men, the mandible, and the most commonly diagnosed and referenced lesion is the PGCG.

The main focus of treatment is excision without tissue curettage with subsequent histopathological study. The lesions show a high recurrence rate that often require explantation, partly due to large bone loss or multiple recurrences. This recurrence rate is not affected by curettage after excision.

Histopathological diagnosis is important, since if the result is a PGCG, the dentist will know that there is a higher risk of bone loss and a higher recurrence rate. Oral hygiene instructions and care by professionals in avoiding leaving "foreign" materials during treatment or rehabilitation should be considered in this entity. These conclusions should be interpreted with caution due to the low number of published cases.

## Data Availability

All data generated or analyzed during this study are included in this published article.

## Abbreviations

PG: Pyogenic granuloma
PGCG: Periapheral giant cell granuloma
PICO: Population, or Problem, Intervention or Exposure, Comparison (if appropriate), Outcome you would like to measure or archieve
